# Non-Targeted RNA Sequencing: Towards the Development of Universal Clinical Diagnosis Methods for Human and Veterinary Infectious Diseases

**DOI:** 10.3390/vetsci11060239

**Published:** 2024-05-26

**Authors:** Stephen Spatz, Claudio L. Afonso

**Affiliations:** 1 Southeast Poultry Research Laboratory, Agricultural Research Service, United States Department of Agriculture, 934 College Station Road, Athens, GA 30605, USA; stephen.spatz@usda.gov; 2 BASE2BIO, 1945 Arlington Drive, Oshkosh, WI 54904, USA

**Keywords:** next-generation sequencing, NGS, clinical diagnostics, metagenomics, non-targeted, RNA, agnostic diagnostics, veterinary, human

## Abstract

**Simple Summary:**

With advances in NGS technology, the high ratio of throughput/cost per base has allowed research laboratories to develop diagnostic protocols that can detect multiple agents (metagenomics) simultaneously without a previous hypothesis. This has created a shift from hypothesis-based diagnosis to agnostic–diagnostic and a clear awareness of how the interplay among traditionally disease-causing agents, opportunistic microbes, and the host itself all influence the host’s health status. However, large-scale implementation of this new technology at the clinical level has not been achieved. We investigated the causes of the implementation impediment and the possible solutions. We examined scientific publications, institutional information, and company sites searching for available research and commercial diagnostics applications of non-targeted sequencing and metagenomics studies focused on the last 10 years. We have identified an extensive range of research applications for this technology across veterinary medicine, human medicine, plant disease, ecological monitoring, and basic biology, all utilizing variations of one very similar general approach. Our analysis revealed that the most significant limitation for clinical applications is operational, stemming from the technology’s high complexity and the lack of validation data. Our analysis suggests that operational research focused on utilizing fewer commonly used technical approaches offers a potential solution. We propose the use of non-targeted total RNA sequencing as the most promising technique. Furthermore, we identified commonalities among existing protocols that could be standardized for validation. To achieve uniformity and validation, we propose consolidating protocols through implementation research guided by existing international organizations and utilizing universal agent-rich reference sample materials.

**Abstract:**

Metagenomics offers the potential to replace and simplify classical methods used in the clinical diagnosis of human and veterinary infectious diseases. Metagenomics boasts a high pathogen discovery rate and high specificity, advantages absent in most classical approaches. However, its widespread adoption in clinical settings is still pending, with a slow transition from research to routine use. While longer turnaround times and higher costs were once concerns, these issues are currently being addressed by automation, better chemistries, improved sequencing platforms, better databases, and automated bioinformatics analysis. However, many technical options and steps, each producing highly variable outcomes, have reduced the technology’s operational value, discouraging its implementation in diagnostic labs. We present a case for utilizing non-targeted RNA sequencing (NT-RNA-seq) as an ideal metagenomics method for the detection of infectious disease-causing agents in humans and animals. Additionally, to create operational value, we propose to identify best practices for the “core” of steps that are invariably shared among many human and veterinary protocols. Reference materials, sequencing procedures, and bioinformatics standards should accelerate the validation processes necessary for the widespread adoption of this technology. Best practices could be determined through “implementation research” by a consortium of interested institutions working on common samples.

## 1. Introduction

Metagenomics, the analysis of all microbial DNA in a sample, offers a powerful foundation for revolutionizing infectious disease diagnostics in veterinary and human medicine. Unlike traditional techniques that target specific pathogens, metagenomics can comprehensively identify any organism present, including novel or unexpected ones. This approach boasts high pathogen identification rates and enhanced specificity due to its reliance on next-generation RNA sequencing (NGS). NGS allows rapid sequencing in parallel of vast amounts of RNA, revealing the total transcriptional landscape of microbes in the sample. This functional information guides pathogen identification and treatment selection. However, significant hurdles remain to be solved before the approach becomes mainstream. Here, we describe numerous applications that highlight the enormous potential of the technology in the human and veterinary fields, alongside the key challenges limiting its translation into routine practice. To overcome these obstacles, we propose the utilization of non-targeted RNA sequencing, which sequences all RNA molecules present, and the adoption of common core analysis steps to streamline the process for cost-effectiveness and clinical efficiency.

## 2. Classic Diagnostics versus Metagenomics

Humans and animals are populated by viruses, bacteria, archaea, fungi, and protozoa, all of which contain nucleic acids that can be utilized as targets for agent detection. Currently, classic human clinical, medical, veterinary, environmental, and plant disease diagnosis remains dominated by targeted technologies such as real-time PCR, immunoassays, and growth-dependent microbiological practices. Targeting, in general, refers to the use of specific primers for nucleic acid capture or amplification. Single tests, or in some cases multiple agent-targeted PCR-based tests, are highly sensitive and of low cost but provide a type of positive–negative result that often requires additional tasks for definitive diagnosis. Targeted sequencing is a more specific version of next-generation sequencing (NGS) that focuses on detecting variations in selected regions of DNA or RNA to identify genetic variants or species. It can be used to enrich the sample by capturing desired mRNAs or fragmented DNA or to deplete other RNA of less diagnostic value. A common targeted approach is the study of the hypervariable region in 16S of bacteria or the 18S ribosomal RNA genes and the internal tandem spacer (ITS) sequence for eukaryotic or fungi. This approach is widely used to characterize bacterial or eukaryotic populations based on RNA sequences of the 16S, 18S, or, in some cases, 23S ribosomal RNAs. CRISPR/Cas9 with genome-wide guide RNAs can be used to deplete “contaminating” host genomics sequences [1]. Targeted sequencing has also been widely used during the COVID outbreak to characterize the SARS-CoV-2 genomes [2,3,4]. Lastly, another targeted metagenomics approach that is mainly used for DNA-based organisms is whole-exome sequencing (WES), another variant of NGS that just characterizes the protein-coding regions of the genome. While WES can be useful for DNA-based organisms, it is not ideal for a universal diagnostic method because it misses RNA genomes or non-coding regions that might be important for the identification of unsuspected agents. Targeted methods, in general, require the utilization of primers to conserved regions and thus may not always differentiate between closely related bacterial species and miss viruses altogether. A main disadvantage of targeted approaches is that they require a previous hypothesis and the use of specific reagents.

While classical targeted approaches offer cost advantages, non-targeted direct sequencing methods provide several compelling benefits. A completely random approach offers a better prospect as a universal diagnostic method. NGS metagenomics goes far beyond just whole-genome sequencing (WGS) of a single organism. WGS of the genetic material from the entire microbial community, e.g., bacteria, eukaryotes, and viruses, theoretically enable the identification of genetic changes such as single-nucleotide polymorphisms or larger changes such as insertions, deletions (INDELs), and translocations. Metagenomics has been defined loosely as the non-targeted production of sequencing data from nucleic acids by next-generation sequencing. DNA or RNA molecules are amplified with random primers and sequenced in one of many NGS platforms. The sequencing output is later analyzed through bioinformatic techniques/pipelines. This analysis is crucial for identifying the causative agents of infectious disease. A major advantage of this approach is its high throughput, which allows for the definitive identification of multiple agents, including both pathogens and commensals.

Although there are multiple advantages of non-targeted diagnosis in comparison to targeted diagnosis (defined as primer-specific), some have argued that it will never be able to replace real-time PCR due to the need for low cost and speed in diagnostics. A second disadvantage is that it is prone to contamination from the host genome and the environment during sample collection. A third disadvantage is that in comparison to targeted diagnostics, metagenomics often requires a larger quantity of starting material. Finally, the current lack of skilled personnel and equipment in the clinical microbiology lab is seen as an immediate difficulty of metagenomics. Mitchell, in 2019, highlighted the high complexity of metagenomic diagnostics (e.g., library preparation, instrument knowledge, problem-solving) and suggested a need for a shift in the skillset of the clinical laboratory workforce [5].

Regardless of these limitations, there are opportunities to develop strategies that overcome those problems and maximize benefits with minimal cost. Recent advancements are making low-cost metagenomics more achievable. Some of the disadvantages can be overcome by the high throughput of current sequencers or by adopting laboratory practices that optimize the sample collection and preparation protocols. Theoretically, users should be capable of detecting ever-lowering amounts of nucleic acid by using better kits and longer sequencing runs or sequencers with a larger capacity. These include new kits for amplifying low sample amounts (like single-cell sequencing kits), improved methods for sample collection and preparation, tools to remove host DNA/RNAs (ribosomal depletion kits), the use of appropriate controls, and even the development of more affordable sequencing devices. More recently, Pichler et al. (2023) evaluated protocols for single-sample viral metagenomics using the long-read Nanopore Flongle sequencing device. A single viral NGS analysis based on the more cost-effective Nanopore Flongle sequencing with decreased turnaround time and lower produced sufficient sequencing output to provide sensitive virus detection [6]. Here, we discuss how to achieve additional improvements while keeping costs in mind.

## 3. Advantages of Non-Targeted RNA-Based Metagenomics in Unveiling the Complex Microbial Landscape

Ribonucleic acid (RNA) serves as a ubiquitous informational molecule across all biological domains, ranging from viruses to complex eukaryotes. RNA sequencing (RNA-seq) is commonly defined as the process of indirectly sequencing RNA following an RNA-to-cDNA conversion. RNA-seq of cDNA offers a unique window into ongoing biological processes. By capturing the transient RNA molecules, it reflects the current activity of genes, including those expressed by the host itself and any invading pathogens (viruses, bacteria, fungi, or parasites). RNA detection signifies either ongoing transcriptional activity within the host organism or the presence of RNA genomes. Transcribed RNA molecules are typically short-lived compared to DNA; thus making them a more reliable indicator of ongoing biological activity as they reflect a “snapshot” of the current gene expression. The inherent amplification during transcription results in a high number of RNA copies, making them easier to detect. RNAs, additionally, are relatively richer in information with fewer repetitive and non-coding regions compared to DNAs. Furthermore, different types of RNAs can be easily selected if needed (e.g., single-stranded poly-A vs. total or size fractionated). In contrast to DNA metagenomics, since DNA is more stable in the environment, its analysis might detect remnants from past infections no longer relevant to the diagnosis of the current health status. In summary, theoretically, a random, high-throughput RNA-seq approach could provide a better semi-quantitative assessment of both host and pathogen gene expression during infection.

Beyond current limitations, advancements in RNA sequencing technology hold significant promise. For example, the low stability of RNAs and the need for amplification during cDNA preparation make the process of library construction more complex. To avoid these shortcomings, long-read RNA sequencing directly from RNA molecules dRNA-seq (Direct RNA Sequencing, DRS), which eliminates the errors that occur during cDNA amplification and avoids RNA-RNA chimeras produced by the cDNA process, has been championed by Oxford Nanopore Technologies [7]. Direct RNA sequencing is in its infancy and promises to deliver a quick and reliable alternative method by eliminating amplification steps. While currently dedicated mostly to the analysis of epigenetic modifications of native RNA molecules, the technology is rapidly evolving with advancements in software and methods [8,9,10,11,12,13]. This has already led to applications as diverse as viral/viroid detection in grapevines [14] and to the definition of the soil transcriptome [15]. Moreover, direct RNA sequencing holds promise for rapid viability assessment in various samples. Currently, viability methods rely on growth-based tests, which are slow and often require cell lines or culture facilities, hindering prompt decision-making. Direct RNA-seq, by capturing actively transcribed RNAs, could offer a faster and more informative approach. For instance, in the case of viruses (DNA and RNA), identifying specific signatures of ongoing transcription (e.g., RNA modifications, splicing polyadenylation, and capping structures) within tissue samples could serve as evidence of active viral activity, indicating viability. Recently, the U.S. National Academics of Science, Engineering, and Medicine (NASEM) proposed a 15-year initiative to study and catalog the vast array of RNA sequences within cells (the RNome) using dRNA-seq (https://www.science.org/content/article/rna-deserves-its-own-massive-counterpart-human-genome-project-researchers-argue, accessed on 9 May 2024).

As the critical link between microbial communities and human health becomes increasingly recognized, non-targeted analysis using RNA-seq (NT-RNA-seq) is rapidly emerging as a powerful tool for disease diagnosis. The results of this approach, also called agnostic diagnostics or “diagnostics without a hypothesis”, offer a powerful opportunity for more accurate disease management. This analysis of the entire pool of RNA molecules within a sample enables the simultaneous detection of a wide range of potential pathogens. Unlike traditional methods that require prior knowledge of a specific culprit, total RNA-seq can identify not only actively transcribed RNA of currently infectious agents but also non-transcribed viral genomes that may contribute to the etiology of the disease. Unlike targeted real-time PCR tests or classical diagnostic methods, this comprehensive analysis provides a much richer picture of the microbial landscape associated with the disease, which is particularly valuable in complex infections involving multiple organisms or those with unknown effects.

A non-targeted total RNA-based sequencing approach to detect all or selected forms of RNA (e.g., genomic, spliced, nascent, polyribosome-associated, circular RNA, linear noncoding, etc.) may be desirable for some diagnostic applications. There are different technical approaches to conducting high throughput NT-RNA-seq, such as random total RNA sequencing, ribosomal sequencing, RNA viral metagenomics, messenger RNA sequencing (mRNA), long non-coding, short non-coding, and dual RNA sequencing. However, most RNA-seq experiments involve the random amplification of all cDNA molecules after they are generated without any previous selection. These enriched cDNA molecules are then prepared for sequencing using massively parallel deep sequencing technology. The advantages and multiple human, animal, and environmental applications of NT-RNA-seq have been described in many publications [16,17,18,19,20,21,22]. The methods, in general, involved converting RNA (total or fractionated, such as poly(A)+) into cDNA libraries using random primers, followed by deep sequencing. Short sequence reads (100 to 250 nucleotides) are compared to reference databases and clustered into taxonomic units that identify the causative agent.

Traditional disease diagnosis often relies on Koch’s postulates, a set of criteria to establish a single microbial culprit. However, groundbreaking studies like those by Forbes et al. (2016) and Vonaesch et al. (2018) challenge the universality of these postulates for complex infectious diseases [23,24,25]. Forbes evaluated numerous studies that present evidence that the human gut microbiota is a diverse ecosystem constituted by multiple types of bacteria, which are essential for the health of the host. Conversely, disruptions in this gut microbial community composition, known as dysbiosis, have been linked to immune response anomalies. Vonaesch and Antonelly (2016) agree that the “traditional Koch postulates” may not always be valid for studying complex infectious diseases and suggest a more complex reinterpretation of Koch’s postulates, named “ecological”, may be needed [26]. This paradigm shift underscores the need for diagnostic methods like NT-RNA-seq, capable of identifying multiple pathogens in biofilms or those increasingly relevant in polymicrobial diseases (e.g., scarlet fever in humans, post-weaning multisystemic wasting syndrome (PWMS) disease in pigs, runting and stunting syndrome in chickens, etc.), or diseases associated with microbial gut dysbiosis [27,28,29,30].

The complex interplay between microbes and hosts in multi-organismal diseases demands more comprehensive diagnostic methods. Dual RNA-seq holds promise as part of the solution for tackling this challenge. Unlike traditional methods that often target specific pathogens, dual RNA-seq, also called comparative RNA-seq, simultaneously detects and analyzes the total RNA from both the host and any microbes present in a sample [31,32,33]. Evidence from multiple studies suggests that integrating total NT-RNA-seq with data on host responses (immunity, pathology), clinical findings, and host transcriptomics can significantly improve etiological diagnosis. For example, bacterial pneumonia often follows influenza infection as secondary infections involving selected bacteria groups, such as Streptococcus pneumoniae and pyogenes, Staphylococcus aureus, and Haemophilus influenzae. Similar synergistic interactions between bacteria and viruses that are observed in the respiratory tract also occur in the gastrointestinal tract, where bacteria interact directly with enteric viruses.

Despite its promise, dual RNA-seq, which requires the sequence reads to be mapped to host and suspected agents or pathogens, has not yet achieved its full potential as a diagnostic method. As two types of RNA levels are analyzed simultaneously, the process of quantifying reads is more critical than during regular diagnosis. While currently more complex and exploratory than traditional diagnostics, dual-RNA-seq has demonstrated application in parasite, fungal, bacterial, and viral infections. Westerman, in 2017, reviewed the use of transcriptomics as a tool for understanding host changes that accompany bacterial infections of eukaryotic cells [33]. Notably, dual RNA-seq was employed to characterize the human airway epithelium’s response to both commensal bacteria populations with bacterial/viral co-pathogens. While the technology is currently quite complex for routine diagnostics, with most studies being exploratory, the potential of dual RNA-seq unraveling complex infections is undeniable [34]. Another potential application of dual RNA-seq lies in detecting early host response signatures that might predict patient outcomes [34,35,36]. Yadav attempted to identify markers of early infection during dengue infection. For in-depth technical details on bacterial RNA sequencing and bioinformatic analysis, the work by Marsh provides valuable insights [37,38]. The authors not only utilize dual RNA-sequencing but also offer a comprehensive review, outlining the bioinformatic analyses with existing software tools. Some of the tools they discuss include those for host–pathogen read separation, de novo transcriptome assembly for unknown pathogens, and differential gene expression analysis to identify key host and pathogen responses.

## 4. Non-Targeted Nucleic Acid Sequencing as a Universal Diagnostic Approach across Animals, Plants, and the Environment

The widespread adoption of total NT-RNA-seq protocols for diagnostics is strongly supported by the large research interest and the growing body of scientific publications ranging from applications in species as diverse as plants to animals. These studies encompass diagnostic applications across the veterinary, human, environmental, and basic biological fields. The ability to detect multiple infectious agents in diverse types of hosts, such as plants, fish, chickens, swine, bovine, horses, and domestic pets, is a testament to the versatility of this approach. In all these cases, minor variations in a highly similar methodology further demonstrate the robustness and adaptability of NT-RNA-seq as a diagnostic tool and suggest a strong “proof of concept” for the technology. The versatility of RNA-seq for studying host–pathogen interactions extends as far as the plant kingdom. Naidoo et al. (2018) employed this technique to elucidate plant–pathogen interactions [39]. Furthermore, Di Gaspero et al. (2022) evaluated the sensitivity and specificity of high-throughput RNA-seq in plants for classifying infected and uninfected samples of Vitis vinifera [40]. Similarly, in aquatic organisms, RNA-seq meta-transcriptomic studies have been used to reveal the presence of viruses, bacteria, and host–pathogen interactions, respectively [41,42]. Ample work using RNA-seq for active chicken poultry farm surveillance has been recently reviewed by Afonso (2023) with the detection of most viral and bacterial species in respiratory, cloacal, and tissue samples [43]. In these studies, a plethora of DNA and RNA viruses were identified, including sicinivirus, infectious bronchitis virus, infectious bursal disease virus, avian orthoavulavirus, avastrovirus, rotavirus A, influenza A virus, megrivirus, avian metapneumovirus, rotavirus D, rotavirus F, avian orthoreovirus and gallivirus, plus gyrovirus, phacovirus, fowlpox virus, gallid alphaherpesvirus 2, chicken picornavirus 1, chicken picornavirus 5, fowl aviadenovirus D, rotavirus G, and tremovirus A. Similarly, key disease-causing bacteria were also identified in these studies by random sequencing of the ribosomal RNAs present in clinical samples. The bacteria included Enterococcus cecorum, Gallibacterium, Streptococcus pluranimalium, Enterococcus faecalis, Bordetella avium, Enterococcus faecium, Ornithobacterium rhinotracheale, Campylobacter, Salmonella enterica, Avibacterium, Enterococcus hirae, Enterococcus durans, Mycoplasma synoviae, Mycoplasma gallisepticum and Streptococcus gallolyticus among others.

In addition, chicken meat, feces, and dust samples were also analyzed using RNA-seq to detect viruses [44,45,46]. In large production animals, Boros et al. collected swine feces, sera, rectal, or nasal swab samples for studying the prevalence and genetic diversity of a picornavirus in farms and Létourneau (2023), utilized a broader metagenomics approach to identify viruses of the Herelleviridae, Microviridae, Myoviridae, Podoviridae, and Siphoviridae families [47,48]. In cattle, researchers have employed RNA-seq to study bovine reproductive, gastric, and respiratory diseases [49,50,51,52,53]. For cats, Fried et al. (2021) utilized a combination of random metagenomic and transcriptomic analyses to investigate the association between feline calicivirus and feline chronic gingivostomatitis [54]. Similarly, Moreno et al. (2017) characterized the canine fecal virome in healthy dogs and dogs with acute diarrhea using shotgun metagenomics and found bacteriophages comprised 99.8% of the total reads (11,157 contigs and singletons), and the rest belonged to viruses in the order Caudovirales and Microviridae [55].

## 5. The Universality of Applications for Nucleic Acid-Based Non-Targeted Diagnostic Sequencing in Human Health

Metagenomics is transforming our understanding of the intricate relationship between microbes and human health. Instead of being a technique that only identifies a single pathogen, the entire pool of RNA molecules comprising a microbial community is analyzed, which offers numerous valuable applications. It is valuable in identifying causative agents for various conditions, including respiratory illnesses, gastric problems, reproductive tract infections, encephalitis, and sepsis, even if the culprits are difficult or impossible to cultivate using traditional methods. Moreover, the reach of NT-RNA-seq extends beyond specific diseases. It allows researchers the ability to identify microorganisms residing in various body sites, including the skin, nervous system, blood, dental areas, and the mucosa of the genital and reproductive tracts. This unveils a deeper understanding of the complex interplay between microbes and human health in different bodily environments.

The following types of studies encompass a broad spectrum of human infections, both chronic and acute. They exemplify the value of investigating nosocomial (hospital-acquired) and post-surgical infections for the discovery of new or emergent pathogens during outbreaks and the evolution of these viruses over time, thereby informing strategies for prevention and treatment. For instance, Kong, in 2023, explored the potential role of RNA viruses and bacteria in Crohn’s disease by studying stool samples using RNA sequencing [56]. In the context of women’s health, Arroyo et al. (2021) investigated the human cervix microbiome using both DNA and RNA metagenomic sequencing and found that RNA sequencing of cDNAs detected a broader range of microbes compared to that found using DNA sequencing [57]. This suggests that RNA-seq may be a more sensitive method for detecting viruses and bacteria. The respiratory tract is another area where NT-RNA-seq offers valuable insights. Lanaspa, in 2017, reviewed studies highlighting the complex and dynamic nature of the respiratory microbiota in the lower respiratory tract, emphasizing it as an ever-changing ecological niche [58]. Building on this understanding, Edgeworth (2023) described rapid diagnostic workflows based on respiratory metagenomics [59]. These workflows, developed through studies on the 2019 coronavirus pandemic, can deliver results within a mere 6 h. This rapid turnaround time has the potential to significantly impact treatment decisions, allowing for swift escalation or de-escalation of therapies as needed.

Beyond treatment decisions, NT-RNA-seq has other applications. In zoonotic diseases, non-targeted RNA sequencing in parasitic and viral respiratory diseases was presented by Wylezich in 2021, who described the development of a targeted method called VirBaits for the detection of 35 viruses [60,61]. The human gut microbiome is another area where NT-RNA-seq has proven valuable. Non-targeted sequencing for gut diseases was presented by Peterson and others in 2002, who demonstrated excellent sensitivity and specificity for all pathogens tested in stool compared to culture and PCR [62]. Recently, He, in 2023, described the diagnostics value of the application of metagenomic NGS for infections in critically ill patients [63].

Non-targeted total RNA-seq for neurological and neuropathological diseases has been presented by several authors. Saha et al. (2018) utilized RNA-seq-based metagenomic sequencing to investigate the causes of pediatric meningitis and identified the presence of the neuroinvasive Chikungunya Virus alongside other pathogens [64]. This study highlights the potential of RNA-sequencing of cerebrospinal fluids that could complement conventional diagnostics to identify etiologies of meningitis. Wilson et al. conducted a study comparing conventional diagnosis with metagenomics NGS in 204 patients with meningitis and encephalitis. This approach identified additional agents in 22% (13 patients) compared to conventional testing, highlighting its potential to improve diagnostics [65]. Using both cerebrospinal fluid and serum samples, RNA sequencing holds promise for detecting a wide range of viral pathogens in children with meningitis and encephalitis. Studies by Hasan et al. (2020) and Fan et al. (2023) concluded that the large diversity of pathogens causing infections, which greatly outnumbers the available types of tests, is one of the reasons why the etiology of many central nervous system infections remains unidentified [66,67].

A notable example of a neurological disease impacted by metagenomics RNA-sequencing is poliomyelitis, a disabling and life-threatening disease caused by poliovirus, an enterovirus within the family Picornaviridae. In the relentless fight against polio, significant breakthroughs in detection and surveillance methods have occurred over the past years. Traditional detecting methods for poliovirus relied on virus isolation and, more recently, RT-PCR. These methods are labor intensive, significantly hindering a swift response to potential outbreaks. To eliminate this bottleneck, Shaw et al. used nanopore sequencing to directly analyze the viral RNA of poliovirus strains in stool and environmental samples within a remarkable timeframe—under 3 days [68,69]. This swiftness not only reduced detection time but also allowed for the differentiation between various poliovirus strains, including the vaccine-derived strains. This was a dramatic improvement over traditional approaches, allowing public health officials the ability to rapidly pinpoint potential outbreaks and implement effective containment measures before the virus had a chance to spread widely. Similarly, Lizasoain et al. implemented wastewater surveillance to uncover a broader range of other circulating enteroviruses, providing valuable insights beyond those obtained solely from reported clinical cases [70]. Fernandez-Garcia et al. and Klapsa et al. further employed unbiased metagenomics approaches to investigate specific polio outbreaks in Europe by characterizing and tracing the origin of the viruses to Africa [71,72]. These techniques were vital for understanding transmission patterns and implementing swift public health responses, including increased surveillance and vaccination campaigns to contain the outbreak. Finally, a recent resurgence of polio in the United States, reported by Russo et al., underscores the ongoing need for metagenomic RNA-seq and robust surveillance programs [73]. This case, the first of paralytic poliomyelitis in the US in nearly a decade, was caused by a circulating vaccine-derived poliovirus type 2. This incident serves as a stark reminder of the potential consequences of declining vaccination rates. In conclusion, the emergence of nanopore sequencing offers a game-changing tool in the fight against polio. Its speed, accuracy, and ability to directly detect the virus hold immense promise for improving global polio detection and eradication efforts.

Non-targeted RNA-seq offers vast potential across many distinctly different fields that apply to human health. Studies by Smibert et al. (2020) suggest that anaerobic commensals are associated with T-regulatory cells, and the microbiome may play a role in allogeneic stem transplant, while Ichiyama et al. (2019) linked bacterial profiles to success rates in embryo transfer [74,75]. In sepsis diagnosis, Wang et al. (2023) demonstrated the superior detection rate of viruses and anaerobes in skin and soft tissue infections by metagenomics-NGS compared to blood culture [76]. Public health surveillance can also benefit from non-targeted applications of RNA sequencing. Stockdale et al. (2023) demonstrated its potential for monitoring COVID-19 and other viruses in untreated wastewater [77]. This approach can provide early warnings of outbreaks and inform public health interventions. Similarly, Santiago-Rodriguez and Hollister (2020) described its use in detecting viruses in settings with high sample volumes, such as schools and hospitals, or in procedures like fecal transplantation, where virus transmission is a concern [78,79]. Liang and Bushman (2021) suggested evidence that the status of the viral community is indicative of health or disease in humans and proposed the concept of a “viral dark matter” due to the vast number of uncharacterized viruses in the human body [80]. Modha, using a machine-learning-based virus prediction tool, DeepVirFinder, reported that about 50% of all uncharacterized sequencing contigs in metagenomics studies are predicted to be of virus origin. In summary, there are countless scientific applications for NT-RNA-seq; however, there are very few adopted protocols for general use in the clinical setting.

## 6. The Paradox of Non-Targeted NGS Applications for Clinical Diagnosis

The paradox of NGS metagenomics is that until recently, many authors have considered that the technology was not ready for general use. Amrane and Lagier, focusing on the limitations of metagenomics for data interpretation and application in routine clinical microbiology, concluded that while NT-RNA-seq holds promise, the technology was not yet reliable enough for general use in clinical microbiology [81]. The key problem is that with the existence of a vast array of technical options at each step of data production, large flexibility is created, producing a seemingly endless continuum of information and data points, which can ironically limit its overall reliability. The outcomes are normally a succession of diverse techniques, steps, and technologies that have not yet matured to be standardized. The fact that sample preparation methods have multiple opportunities for modifications, combined with the fact that each modification is likely to affect the outcome, makes it very difficult, and, in some cases, nearly impossible to obtain identical metagenomic results from different laboratories. Furthermore, in this continuum of options, clinical doctors are not properly trained to evaluate protocols or to understand the consequences of each change on the outcome, and, in most cases, they are unable to correctly interpret the data. A similar situation occurs in both the human and the veterinary fields. A spectrum of results that are highly affected by the experimental variables may not be helpful to doctors, veterinarians, and officers in the fast-paced clinical setting. Clinicians desperately require clear, positive–negative results to guide their decisions. Unfortunately, the complexity of metagenomic data can make such definitive answers elusive, leading to indecision. The consequence is that doctors and veterinarians may select a more limited type of diagnosis, albeit a less precise diagnostic method, because “at least it is operational”.

Standardization remains a significant hurdle in metagenomics. This dilemma needs to be addressed before non-targeted NGS can be widely adopted in medical and veterinary clinical practice. Recently, the challenges and prospects of clinical metagenomics in medicine were reviewed by Batool [82]. The authors hail metagenomics sequencing as a promising culture-independent technology. This method has no preconceived biases, making it ideal for situations where diagnosing the precise cause is challenging due to rare, uncharacterized pathogens or even co-infections. However, they recognize the need to resolve the shortcomings in terms of sensitivity, specificity, costs, standardization of bioinformatic pipelines, and interpretation of findings before NGS metagenomics can be integrated into clinical practice. The author acknowledges the potential for NGS metagenomics as a clinical diagnostic tool for identifying severe infections but emphasizes the need to conduct large-scale clinical and cross-institutional studies to validate its efficacy. Batool also highlights the impact of various factors such as sample type, quality of sampling, the platform used, number of reads generated, and data analysis tools on the sensitivity and specificity of NGS metagenomics. It was concluded that it was unlikely that metagenomics may be utilized as a first-line approach unless those issues are resolved, perhaps leaving metagenomics-NGS as a last resort for time-sensitive diagnostics in life-threatening infections.

## 7. The Need to Add Operational Value to NT-RNA-Seq

The above limitations of NGS metagenomics in the clinical setting can be summarized as “of restricted operational value”. Operational value could be defined as the “usefulness or utility”, meaning that the data generated may not translate into clear, actionable reports that inform treatment decisions for patients. Operational value could also be interpreted as “validation”. This is the process that determines the fitness of a test for an intended purpose. It is expected that any diagnostics test be validated for the different species and diagnosis needs. This validation process goes beyond simply determining if a test works. It assesses the test’s performance across a range of applications crucial for animal health, including surveillance programs to determine freedom from disease in farms, regions, and even entire countries; confirming a diagnosis in individual animals suspected of infection; estimating the overall prevalence of a disease within a population; identifying infected animals within herds to implement appropriate control measures; evaluating the effectiveness of vaccination programs, etc.

Among the challenges to creating operational value, one problem is the lack of training, and the other is the absence of standard operating procedures. Graff and others, in 2021, evaluating human metagenomics for diagnosis in humans, identified gaps in education and in the clinical guidelines hindering the implementation of NGS-based metagenomics in diagnosing pediatric meningitis and encephalitis [83]. They found that variability across indications, patient populations, and time of testing significantly impacted the clinical utility of the technology. This study highlighted the importance of training and clinical guidance for the best use of this technique and cost-effectiveness stewardship in timing and interpretation of negative results.

Building on previous work, Graf recently delved deeper into the challenges of utilizing NGS for clinical metagenomics [84]. Despite unanimous agreement on the immense potential and need for standardization of a pan-pathogen detection test, key questions remained. A crucial one was defining the clinical scenarios where this test would be most beneficial as a first-line diagnostic tool, along with the most appropriate specimen types for analysis. Part of the issue has been the lack of agreement over cost–benefits. Other limitations arise from variations in clinical sensitivity and differences in specificity among laboratories. Laboratory differences could be related to sample sources, sterile culture sources, contaminating organisms introduced during the pre-analytical process, or differences in analyzing and reporting approaches that may exclude/include contaminating flora or latent viruses [85]. To overcome these hurdles and achieve true clinical utility, these authors emphasized the need for “more guidance” on reports and further optimization and standardization across the entire workflow, encompassing pre-analytical steps (specimen selection) through interpreting the role of potential contaminants such as contaminant flora.

## 8. Need for Identification of the Best-Use Practices That Accelerate Implementation

In the past, as illustrated above, a large emphasis on metagenomics has been put on developing new applications and methods for diagnostics; however, little effort was directed toward investigating the best-use practices that could improve operational value for individual applications. It can now be argued that many of the issues limiting the widespread adaptation of NGS metagenomics are implementation-related, suggesting that these problems are not insurmountable. A crucial step forward would be establishing standardized implementation strategies across the field. In a recent article, Gaston (2023) described the challenges and progress toward producing the operational value of clinical metagenomics for infectious diseases [85]. As highlighted by the author, the core focus lies in leveraging clinical metagenomics to provide actionable results that directly benefit patient care. In human medicine, the stakes are higher as the lives of patients are on the line. This necessitates stricter regulations to ensure safety and efficacy.

Gaston further describes the existence of numerous challenges hindering the operational value of NGS metagenomics to achieve results. These challenges span the entire workflow, from sample acquisition and preservation to complex bioinformatic analysis and interpretation. Moreover, each step presents its own set of variables, including various extraction protocols, enrichment techniques (e.g., host nucleic acid depletion, amplification, target capture), library preparation (with potentially multiple types of kits or reagent options), sequencing platforms, and bioinformatic tools. The need to integrate information from multiple databases and software and difficulties communicating actionable results further add to the complexity. However, Gaston also identifies opportunities for improvement. Firstly, to develop strategies that would add operational value for clinical use by identifying commonalities across existing protocols with the intent of adopting standardized operation procedures (SOPs) that maximize the benefits of NGS metagenomics and, secondly, to accelerate the unification of approaches that are common to all types of diagnostic tests. These objectives can be achieved through implementation research so that NGS metagenomics can be integrated into routine clinical practice.

### 8.1. Identification of Commonalities as the First Step toward Implementation Research

**Figure 1:**
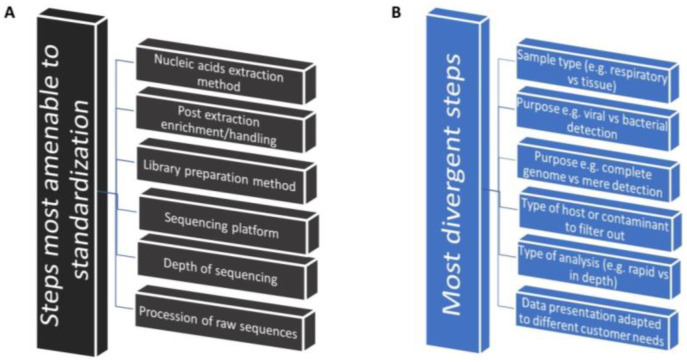
Amenability of NT-RNA-Seq steps to standardization across different types of applications. (**A**) Most common steps. (**B**) Most divergent steps.

There are significant technical commonalities across most of the applications described above. Identification of commonalities is the first step toward creating common standard operating procedures to be tested via implementation research. The first key technical commonality emerges in the use of total RNA as the substrate for metagenomics in infectious disease diagnosis. The benefits of the use of total RNA are described above. This preference for RNA lies in its superior ability to capture actively transcribed pathogens, e.g., from DNA-based microorganisms, or directly to detect the RNA genomes of viruses and viroids. Significantly, additional commonalities exist in most of the subsequent steps, including nucleic acid extraction, processing, host background depletion, sequencing, bioinformatics analysis, and even the potential application of artificial intelligence (Figure 1 shows the most common and the divergent steps for non-targeted NGS standardization). These shared elements present a valuable opportunity to standardize protocols across the field, streamlining the entire diagnostic process.

Commonalities are most abundant in certain core parts of the protocols (Figure 1A) but less abundant at the initial (sampling) and the final steps (data analysis and interpretation) of the process (Figure 1B). The sampling strategy is particularly important for non-targeted sequencing since the sampling methodology is not likely to be identical for all different applications. In the past, for targeted diagnostics, sampling has not been that critical (e.g., PCR and real-time PCR) because amplification using specific primers minimizes problems caused by environmental and host contamination. In contrast, NT-RNA-seq analyzes the entire RNA profile, making optimal sample selection crucial for accurate results. The choice of tissue or organ for sampling significantly impacts the type and levels of contaminants. For instance, respiratory samples are highly different from cloacal samples in terms of the levels of bacterial contamination and the diversity of co-sampled microbes. Bacteria in cloacal samples, crucial for studies involving gastric problems, may not be as critical for their detection in respiratory samples. Incorrect sampling techniques can be used if the method used disrupts the mucosal tissue and inadvertently collects large amounts of contaminant host cells. Thus, avoiding host tissue contamination may or may not be important in some cases, depending on the purpose of the assay. Conversely, sequencing at a deeper depth or using techniques like bacterial RNA depletion might be necessary for sensitive viral detection in these samples, where viral RNA is less abundant (Figure 1B). Similarly, the presence of tissue-specific viruses in some animal secretions may necessitate deeper sequencing or make it necessary to deplete host ribosomal or bacterial RNA to improve detection. These examples highlight the significant influence that choosing the correct sampling protocols may have on RNA-sequencing strategies. Thus, the development of standardized protocols should prioritize procedures designed according to sample type (respiratory, cloacal, tissue, blood, etc.) regardless of the host (Table 1, critical aspects of sample collection).

Because cost-effectiveness may play a crucial role in sampling strategies for non-targeted NGS, it is important to have a fit-to-purpose approach tailored to address a specific diagnostic question. Pathologists and veterinarians need to carefully design sampling strategies to optimize non-targeted NGS results while remaining fiscally responsible (Table 1). Multiple publications have described the challenges and common protocols associated with sample collection and preparation in this context. Contamination during sample collection has also been addressed by several authors [86,87,88]. Bunholi, in 2023, addressed the critical need for methodological standardization in environmental DNA and RNA analysis applied to aquatic communities [89]. Their study reviewed 291 papers and concluded concerns about methodological inconsistencies. This variability spanned factors such as water filtration volumes, filter material, filter pore size, extraction method, marker choice, and bioinformatic pipelines. They emphasize that standardization is essential for facilitating comparisons across different aquatic systems and ultimately enabling the broader integration of NGS applications in biodiversity monitoring efforts. Kurian et al. (2020) conducted comparative studies on fecal collection methods in microbiome studies involving solid organ transplant recipients [90]. Their study compared fecal swabs and wipes to the traditional scoop methods using 16S ribosomal RNA sequencing and shotgun metagenomics to assess bacterial abundance and diversity. The results revealed that swabs are the preferred alternative due to their resemblance to the scoop method’s result and their ease of collection and processing compared to the wipes. Maghini et al. (2023) investigated how sample condition, preservative method, and bioinformatic analyses can introduce bias in microbiome measurements, impacting both relative and absolute abundance data [91]. As a result, they recommend the use of preservatives for field studies and commercial DNA/RNA protection products for metatranscriptomics studies, followed by absolute quantification for microbial measurements. Sample preservation and storage, shipping, and extraction are likely to significantly influence results. Afonso’s 2023 review also provides a valuable resource outlining critical aspects of sample collection (Table 1) [43].

**Figure 2:**
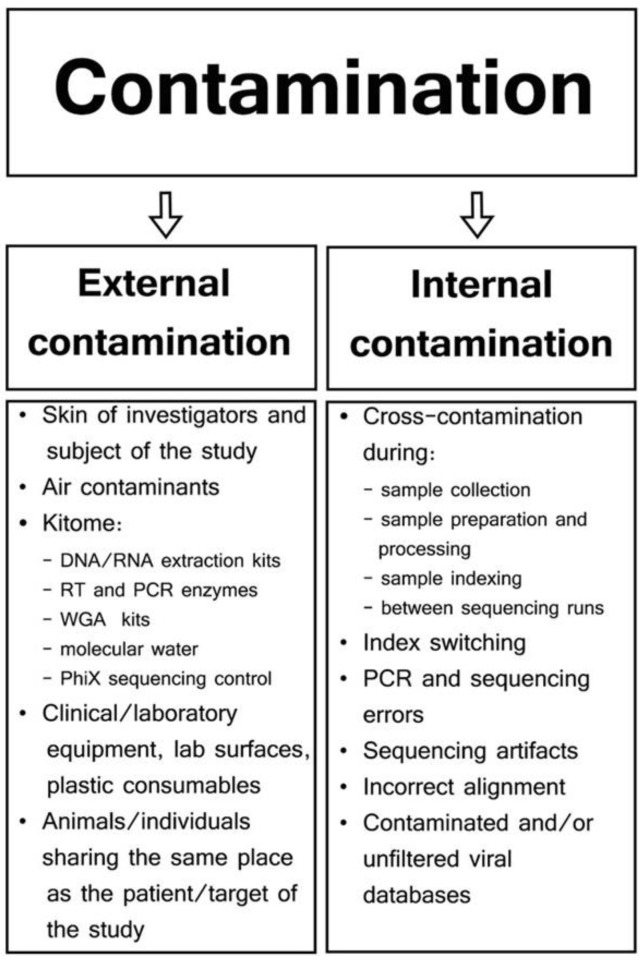
Types and sources of contamination in viral metagenomic studies.

Standardization seems most achievable for several key steps in RNA-seq workflows, including nucleic acid extraction, post-extraction nucleic acid handling, library preparation, sequencing platforms, and initial processing of raw sequence data (Figure 1A). A comprehensive analysis of existing methods and results should be the first step in identifying shared features or attributes among divergent types of experiments. Technical commonalities in nucleic acid extraction, library preparation, and sequencing are easier to address for these steps and could be targeted immediately. There is a vast amount of literature on total RNA-seq methods, providing a strong foundation for this endeavor [92,93,94,95,96,97,98,99,100]. Comparative studies have already begun to identify commonalities across different experimental procedures. However, key challenges remain, such as internal contamination or contamination during sample processing. Jurasz, in 2021, specifically addressed the issue of contamination in viral metagenomics, which is graphically shown in Figure 2 (adapted from Jurasz, H.; Pawłowski, T.; and Perlejewski, K., *Contamination Issue in Viral Metagenomics: Problems, Solutions, and Clinical Perspectives*, in *Frontiers in Microbiology* [87]). 

Another area of advancement is ribosomal RNA (rRNA) depletion. A major hurdle in RNA-seq for viral detection is the presence of ribosomal RNA (rRNA). Comprising 80–95% of the cell’s RNA, cellular ribosomal RNAs are normally one of the most serious contamination problems since they can mask the much smaller signature of viral RNA in tissue samples. Removal of host RNAS with biotinylated probes designed for specific hosts can be used in conjunction with RNase H to degrade the rRNA before library prep [101,102]. Culviner et al. (2020) described a cost-effective and robust method for rRNA depletion in RNA-seq studies [103]. Their approach utilized biotinylated oligonucleotides that targeted bacterial rRNAs (23S, 16S, and 5S) and physically removed the bound rRNAs before sequencing with magnetic streptavidin-coated beads. Strategies for the amplification of RNAs for sequencing, which has some potential to introduce biases, can be improved upon by adding RNA spike-ins to samples. These artificial pieces of RNA are normally a mixture of synthetic RNA transcripts of known sequence, concentration, and abundance. The spike-in sequences should be unique and different from both the host and pathogens of interest [104].

Several recent studies demonstrate similarities in approaches and significant progress in optimizing methods for viral detection using NT-RNA-seq [95,96,97,98,105,106,107]. Yang et al. (2022) employed a metagenomic approach in pigs to identify circulating viruses [108]. Samples were collected in phosphate-buffered saline (PBS), homogenized, filtered, and treated with either DNase or RNase before viral RNA and DNA were extracted and sequenced. The viral metagenomic approach identified viruses belonging to the families Anelloviridae, Arteriviridae, Astroviridae, Flaviviridae, Circoviridae, and Parvoviridae; prokaryotic virus families including Siphoviridae, Myoviridae and Podoviridae. Ogunbayo et al. (2023) investigated the impact of different sample preparation techniques on the respiratory RNA viruses from nasopharyngeal swabs [97]. Their study compared various extraction kits, DNase treatments, and rRNA removal methods. As expected, all these factors—sample collection, extraction, and enrichment strategies—significantly influence the detection of respiratory viruses.

Automation and microfluidics for metagenomics can offer significant advantages, but each has its limitations. Some of the advantages of automation include (i) increased throughput in which a larger number of samples are processed, leading to faster analysis; (ii) reduced variability due to minimizing human error, which can result in more consistent and reliable data; and (iii) reduced contamination risk by the operator and the environment. However, automated systems are initially expensive with costly proprietary consumables and might not be easily adaptable to handling diverse samples, thus limiting flexibility. Additionally, there is some potential for failures when using small sample sizes that require extensive amplification. Microfluidic devices can alleviate this since these devices require significantly smaller sample volumes, making them ideal for analyzing precious or limited samples. This miniaturized workflow combined with implementation testing may reduce the risk of failure while keeping reagent costs and human-caused contamination down. Choosing the appropriate approach depends on the specific needs of the research, considering factors like sample availability, desired throughput, and budget constraints.

Technical commonalities in bioinformatics and data analysis are another area that could be easily addressed. The fields of metagenomics and metatranscriptomics are rapidly developing, with researchers comparing different tools and protocols for specific applications. Researchers have emphasized the importance of collective efforts to standardize bioinformatics protocols and create more comprehensive datasets with detailed host metadata. This need is underscored by the work of Ibañez-Lligoña et al. (2023), who analyzed existing bioinformatic tools for NGS-based metagenomics analysis in the context of improving the clinical diagnosis for emerging diseases [109]. They concluded that despite the availability of diverse analytical tools for every stage of the metagenomics analysis pipeline, including quality control, pre-processing, filtering out untargeted sequences, assembly, and taxonomic profiling, a single, universally accepted approach remained elusive. A recent article by Gihawi et al. (2023) describes the critical role of good quality control measures in metagenomic analysis [110]. The author outlined key considerations for study design and best practices for QC throughout the workflow.

Several recent studies describe the ongoing development of bioinformatics pipelines that are crucial for advancing metagenomic analysis. Gemler et al. (2023) introduced the UltraSEQ pipeline and compared its performance to several commercially available options. They evaluated these pipelines using publicly available clinical datasets from various samples and data formats, including short- and long-reads [111]. This study exemplifies the efforts underway to refine and improve bioinformatic tools for metagenomics. Grundy also recently utilized metagenomic sequencing to detect pathogens in archived plasma samples from 254 patients with sepsis in Uganda [112]. Additionally, they conducted a study to compare the performance of qPCR and short-read Illumina sequencing for detecting HIV. The Illumina sequencing data were analyzed using the Chan Zuckerberg ID metagenomics bioinformatics (https://czid.org/, accessed on 9 May 2024), a free cloud-based metagenomic platform, and found qPCR could detect the virus in 69% of samples (sensitivity), while sequencing achieved a slightly higher sensitivity of 70% with 92% specificity compared to qPCR.

The fields of metagenomics and metatranscriptomics are rapidly growing beyond analytical software. The creation and ongoing improvements of specialized databases are abundant, and there is a need for uniformity and validation [113,114,115,116,117,118]. The FDA-ARGOS database [119], the SILVA ribosomal database (https://www.arb-silva.de/, accessed on 9 May 2024), and RDP (https://www.glbrc.org/data-and-tools/glbrc-data-sets/ribosomal-database-project, accessed on 9 May 2024) are important but there is a need to develop a consensus database that would have clinical acceptability. Equally, software such as Kraken2 and others that are associated with databases need to be extensively evaluated [120,121]. Terrón-Camero and others recently compared metagenomics and metatranscriptome tools addressing some of the users’ needs [122]. Software tools like Kraken2 and Bracken are examples of a next-generation microbiome bioinformatics platform that is extensible, free, open source, and community-developed. In 2023, Liebhoff developed a pathogen detection method named Pathonoia that still needs further evaluation [123]. In all cases, establishing protocols and standard operating procedures leading to the validation of methods will be crucial for metagenomics to mature from a research lab tool toward a widely adopted standard clinical diagnostic tool.

Although in its infancy, technical commonalities in artificial intelligence may benefit from identifying the best operational procedures to fit purposes. Artificial intelligence will likely make contributions to bioinformatics analysis and data interpretation. In theory, AI could streamline workflows and potentially reduce the reliance on highly trained personnel. However, AI is relatively new, and the exact nature of its implementation remains uncertain. Some preliminary work has already been conducted, and AI is already showing potential. For instance, Seneviratne et al. (2020) reviewed studies exploring the link between the oral microbiome and systemic diseases from the perspective of artificial intelligence [124]. The author addressed the factors that limit the value of current studies, including variations in study design and the presence of confounding variables. Modes in which artificial intelligence can be employed for predicting systemic disease and host metadata from the oral microbiome were also suggested. Similarly, Zeng, T., in 2021, explored how artificial intelligence can be harnessed to extract valuable data for studying the microbiome for gastrointestinal diseases [125]. Likewise, Baugher et al. (2021) investigated the potential for AI to improve diagnostics in female urinary tract infections. Their study compared the microbiome analysis of patients with urological symptoms versus healthy ones. An AI-powered tool was used for pathogen identification in all the samples. The results using this AI tool were consistent with those provided by infectious disease experts, suggesting its potential for accurate pathogen identification and improved reporting.

However, many AI challenges remain that will require cooperative work among researchers. Gubatan et al. (2021) reviewed existing artificial intelligence applications in inflammatory bowel disease [126]. The authors recognized the potential of AI for analyzing, integrating, and interpreting large metagenomic datasets. However, they highlighted a critical limitation: the lack of robust validation studies with unbiased, prospective designs. They emphasized the need for collaborative efforts among researchers to generate larger datasets with comprehensive host metadata to address this crucial gap. In agreement, Wani and others, in 2022, explored the importance and possibilities of metagenomics and artificial intelligence in the context of human health [127]. The authors stressed the importance of the microbiome in human health and the potential of metagenomics for exploring microbial communities in a culture-independent mode. The authors pointed to the potential of artificial intelligence to add dimensionality to the data and use it to understand its complex relationship with disease. Overall, establishing comprehensive datasets with detailed host metadata remains a crucial component for advancing AI in the metagenomic field.

### 8.2. Comparative Studies on the Implementation

Once the key commonalities have been identified, large-scale comparative studies may be needed to determine which one of the existing technologies or protocols is most appropriate for implementation. The good news is that the standardization of methods and tools and the development of appropriate controls are achievable goals, provided that a concerted effort is made by interested institutions. Considering the large number of applications in key human, veterinary, and environmental fields (described above) and the many technical commonalities, there is a compelling opportunity to establish large groups focused on performing comparative studies, identifying best-use practices, and developing methods of standardization. This action could lead to the type of rapid progress needed to move non-targeted RNA-seq from the research laboratory to the clinical lab.

A key area of focus should be developing methods for direct comparison of detection levels side by side in comparison with established procedures such as those based on real-time PCR. For example, a comparison of results (e.g., number of reads) obtained with random RNA-seq versus well-established methods like single-plex and multiplex real-time PCR could be a valuable avenue toward standardization. Excellent work has been started by creating specialized meetings on virus detection for biologics and vaccines, with conclusions reported in international conferences on next-generation sequencing for adventitious virus detection in biologics [128,129]. There, international experts from various sectors, including regulatory agencies, academia, contract research organizations, biotherapeutics, and biologics industries, discussed standardization and validation of NGS steps for the characterization and safety evaluation of biologics, including vaccines for humans and animals. The group’s conclusions emphasized the potential of NGS for broad-spectrum virus detection, encompassing even uncharacterized viruses. They envisioned NGS complementing, replacing, or working in conjunction with traditional assays for adventitious virus detection.

Highlighting the need for international standards in metagenomics and transcriptomics, Mason et al. addressed the issues surrounding the creation of these standards [130]. Mason proposed leveraging the following organizations to address current challenges in NGS protocols: Sequencing Quality Control Consortium (SEQC) from the U.S. Food and Drug Administration (FDA); the Association of Biomolecular Resource Facilities (ABRF)-NGS group; the Centers for Disease Control and Prevention’s (CDC) Next-Generation Sequencing Standardization of Clinical Testing group; the NIST, National Institute of Standards and Technology (Gaithersburg, MD, USA); iGEM, International Genetically Engineered Machine (Cambridge, MA, USA); EU, European Union; BEI, BEI Resources (Manassas, VA, USA); NIAID, National Institute of Allergy and Infectious Diseases, U.S. National Institutes of Health (Bethesda, MD, USA); Meta, MetaGenoPolis (Jouy-en-Josas, France); Zymo, Zymo Research (Irvine, CA, USA); ATCC, American Type Culture Collection (Manassas, VA, USA). Overall, these organizations, despite their diverse backgrounds, all have vested interests in improving and standardizing practices related to next-generation sequencing (NGS).

The ever-increasing number of non-targeted NGS assays creates a need to implement solutions that allow for the rapid validation of different methods, instruments, protocols, and facilities. To accelerate the process, the Food and Drug Administration (FDA) in January 2024 released the availability of a guidance for the industry entitled “Q5A (R2) Viral Safety Evaluation of Biotechnology Products Derived from Cell Lines of Human or Animal Origin”. This guidance was supported by the International Council for Harmonization of Technical Requirements for Pharmaceuticals for Human Use (ICH). The guidance included includes, among other protocols, the use of non-targeted NGS. Equally, European guidance has recently been released. High-throughput sequencing for the detection of viral extraneous agents (https://www.edqm.eu/en/-/pharmeuropa-36.2-just-released, accessed on 9 May 2024). This document described the requirements for acceptable use of the NGS approach in greater detail. The use of spiking studies or evaluations based on well-characterized validated samples (reference samples) is likely to provide a useful and holistic method of rapid assessment. As deficiencies in some steps in the protocols can be compensated in others steps, giving similar outcomes (e.g., a better amplification step may compensate for lower amounts of RNAs during extraction), a comparative evaluation process based on the use of accepted and universal reference biological material seems to be highly relevant.

The use of universally validated “sample kits” containing representative agents of the different types would allow the evaluation of complete pipelines from sample to report. For example, highly controlled and well-characterized samples of human fecal matter or respiratory secretions could be distributed and used to determine the acceptability of the basic core protocols. These complex universal samples could simplify the validation process making it independent of the protocols. Reputable agencies could discuss the composition of those samples. Thus, it should be possible to immediately determine the acceptance or rejection of protocols based on the sensitivity and specificity performance of an entire process on “representative samples”. Currently, FDA-CBER, USA, is developing reference materials for adventitious virus detection by NGS. https://rvdb.dbi.udel.edu/, accessed on 9 May 2024. The effort was initially focused on utilizing five reference virus stocks for the NGS adventitious virus and on a collaborative study among laboratories to evaluate virus detection by different NGS platforms. Preliminary studies indicate that despite the differences in platform and laboratory practices, the results have been comparable. Specifically, for the NGS bioinformatics analysis, nucleotide versions of viruses databases are publicly available at the University of Delaware (https://rvdb.dbi.udel.edu/, accessed on 9 May 2024) with a link to the protein databases, which are available on the Institut Pasteur (https://rvdb-prot.pasteur.fr/, accessed on 9 May 2024. Similarly, the reliability of agent detection depends on the availability of complete and well-curated databases, the appropriate selection and configuration of sequence alignment programs, the removal of host sequences without loss of sequences of interest, and the selection of a threshold for detection to avoid false positives. Efforts are being conducted by several private companies to compensate for deficiencies in publicly available protocols.

A change in mindset may also be necessary regarding the expectations of the technology. Classical non-targeted NGS has mostly been used to identify disease-causing agents; however, non-targeted NGS may have different beneficial applications. For example, it could be used to identify a host microbial community’s “healthy vs. diseased” states. It could be very useful to establish thresholds of detection for “groups of agents” corresponding to “type of sample”. Categorizing the agents into broad types (e.g., DNA, viruses, RNA viruses, fungus, bacteria, etc.) according to sample types (e.g., human brain, avian cloacal, swine respiratory) and establishing acceptable numbers or agents for “normalcy” may be a possible application of non-targeted NGS in clinical diagnosis. As today’s human blood tests, when levels of enzymes, nutrients, and minerals are abnormal, indirectly indicating healthy or disease status, the identification of normal thresholds of bacterial or viral populations (some commensal and non-pathogenic) is likely to provide predictors of disease outcomes. This type of utilization can only be achieved when standardized testing provides the baselines.

Human clinical samples present a unique challenge in metagenomics RNA-sequencing. In unenriched samples, a large portion of the sequenced reads will originate from the host’s RNA rather than the target microbes. This raises concerns about privacy and HIPAA (Health Insurance Portability and Accountability Act of 1996), as well as unnecessary sequencing costs on a target of noninterest. Fortunately, this may be resolved technically and legally. Several approaches can help mitigate the issue of host RNA overwhelming the microbe data. These include laboratory subtraction methods, bioinformatics subtraction techniques, or simply regulation that may help in this aspect. In summary, the goal is to identify commonalities among widely used protocols followed by rigorous step-by-step validation, including legal issues. Implementation research to evaluate performance across different laboratories and the selection of best-fit SOPs and protocols should pave the way for the faster adoption of universal metagenomic protocols.

## 9. Conclusions

Non-targeted RNA-seq offers several attractive features that make it potentially very useful for a wide number of diagnostics applications. Unlike DNA-based methods, it can evaluate transcriptional active genes in all types of living organisms, including viruses and viroids, making it ideal for developing a universal approach to diagnostics. Because the applications of NT-RNA-seq are versatile, there is wide interest in developing this technology. However, to translate this potential into reality, several existing operational problems need to be addressed. While standardizing sample collection, RNA extraction, processing, sensitivity, and bioinformatic analysis presents challenges, the fortunate decline in sequencing and analysis costs, coupled with automation, could help the process of overcoming these hurdles. Challenges related to bias and the development of standardized testing and operating procedures can be tackled through implementation research across a wide research community. For that purpose, the availability worldwide of validated standard but agent-rich “samples” for validation is likely to be essential. The lack of skilled personnel and equipment in the clinical microbiology laboratory was addressed previously by Mitchell in 2019. He argued that a shift in the clinical lab workforce skillset is necessary because of the complexity of tasks like library preparation, operating new instruments, and troubleshooting issues. However, these earlier predictions likely underestimated the rapid pace of technological advancement and the ease with which skill sets can adapt. Today, it could be argued that those predictions did not fully consider how fast the change in skill sets would occur, nor how many advances are constantly occurring today. The current research landscape, across both academic and private sectors, is yielding an incredible stream of innovations that were unforeseen at the time of Mitchell’s report. Furthermore, some of those skills may no longer be needed as new kits, automation devices, microfluidic devices, and readily available sequencing and bioinformatics services may alleviate technical expertise and equipment limitations. Barriers to implementation, such as reducing the high cost of NGS sequencing and acquisition of technical expertise, are now disappearing with new equipment for sample preparation, robotics, kits, and automation in general. The growing accessibility of bioinformatics resources and databases for end users through online commercial vendors (e.g., www.Base2Bio.com (accessed on 9 May 2024) and Clear Labs (https://www.clearlabs.com/, accessed on 9 May 2024) and QIAGEN (CLC Genomic Workbench, https://www.qiagen.com/us/products/discovery-and-translational-research/next-generation-sequencing/informatics-and-data/analysis-and-visualization/clc-genomics-workbench, accessed on 9 May 2024) further enhances usability. It could be argued that the operational value of RNA-seq will likely strengthen over time as its utility in solving diagnostics problems is repeatedly demonstrated. Notably, commercial applications of metagenomics testing, both DNA- and RNA-based, are already in the market. The University of California at San Francisco has a CLIA-certified clinical microbiology laboratory that offers validated testing for pathogen detection in meningitis and encephalitis from plasma samples or cerebrospinal fluids (https://nextgendiagnostics.ucsf.edu/technology/, accessed on 9 May 2024). This site, showing the potential for real-world clinical usage of metagenomics, offers clinical interpretation reports with a sensitivity of 86.1% and a specificity of 97.9%. With rapid advancements dissolving technical hurdles and unleashing a torrent of innovation, NT-RNA-seq stands poised to revolutionize clinical diagnostics.

## Figures and Tables

**Table 1:** Basic principles of sampling for non-targeted sequencing [43].

*1. Always use “standard operating procedures” for sample-to-sample comparison purposes.*	
*2. Develop “a priori” a sampling strategy focused on the specific problem with the help of a field veterinarian and pathologist.*	
*3. Develop a sampling strategy that covers “completely and evenly” the areas or the host of interest.*	
*4. Minimize contamination from operators, non-target tissues, and the environment at all stages of collection.*	
*5. Minimize post-sampling contamination; use masks, sterile plasticware, media, and antibiotics, if possible, for manipulation and storage.*	
*6. Do not mix different types of samples (e.g., cloacal samples will dilute respiratory samples with bacterial nucleic acids).*	
*7. Obtain sufficient starting sample material (RNA/DNA) to minimize the amplification steps (e.g., pool the same type of samples if necessary).*	
*8. Minimize degradation of nucleic acids (RNAs are very sensitive) by using gloves, cold chains, and RNAse-free reagents.*	
*9. Use trained operators at all stages of the process.*	
*10. Use fast and reliable labeling (printed tags, barcoding, spreadsheets, instead of pens at the site).*	
*11. Obtain and link the most complete metadata possible in all samples (e.g., farm clinical and management information).*	
*12. Note “all’ clinical details associated with the host pathology for each sample.*	
*13. When spotting on FTA cards, rigorously follow the recommendations on expiration dates, spotting volumes, drying time storage, and shipment conditions.*	
*14. Include information in “the shipping form” that will be used for the interpretation of complex results such as:*	
	*Date of collection, the name of the operator, and/or sample contact information.*
	*Flock identification (can be coded for confidentiality).*
	*Type of sample (oropharyngeal, cloacal, tissue).*
	*Species and age of the sampled birds.*
*Optional information: vaccination; suspected disease; clinical lesions; histology; flock health; production problems; GPS location.*	

## Data Availability

No new data were created or analyzed in this study. Data sharing is not applicable to this article.
